# Malignant Syphilis*Medical Bulletin, July, 1898.

**Published:** 1898-08

**Authors:** 


### MALIGNANT SYPHILIS.*

At a meeting of the Society of Internal Medicine of Berlin Dr. Golz presented a young girl who had entered the Moabit Hospital on account of a severe syphilis which resisted all treatment. The ready occurrence of salivation was an obstacle to treatment, and a generalized eruption of partly-ulcerated papules rendered inunction impossible. Injections of sublimate did not prevent the evolution of the disease and particularly of large ulcers of the throat and tongue. The epiglottis was completely lost. An energetic treatment by inunction was, nevertheless, instituted, although a pleurisy developed, the effusion of which was gradually absorbed. Soon afterward a catarrh of the apices of the lungs occurred, but microscopical examination did not show the presence of bacilli in the sputum. Treatment was abandoned in favor of a reconstituent diet, but the body-weight diminished and fell to 76 pounds. Ulcers of the mouth and arthritis of the knee followed, the last named being dissipated by mercurial inunction. The patient soon left the hospital in a comparatively satisfactory condition. Several months later there was not a syphilitic symptom, and the catarrh of the apices had almost completely disappeared. The latter was, therefore, probably, of syphilitic nature.

*Medical Bulletin, July, 1898.

